# State transitions and photosystems spatially resolved in individual cells of the cyanobacterium *Synechococcus elongatus*

**DOI:** 10.1093/plphys/kiab063

**Published:** 2021-02-10

**Authors:** Ahmad Farhan Bhatti, Diana Kirilovsky, Herbert van Amerongen, Emilie Wientjes

**Affiliations:** 1 Laboratory of Biophysics, Wageningen University, Wageningen, The Netherlands; 2 Institute for Integrative Biology of the Cell (12BC), CEA, CNRS, Université Paris-Sud, Université Paris-Saclay, 91198 Gif-sur-Yvette, France; 3 MicroSpectroscopy Research Facility, Wageningen University, Wageningen, The Netherlands

## Abstract

State transitions are a low-light acclimation response through which the excitation of Photosystem I (PSI) and Photosystem II (PSII) is balanced; however, our understanding of this process in cyanobacteria remains poor. Here, picosecond fluorescence kinetics was recorded for the cyanobacterium *Synechococcus elongatus* using fluorescence lifetime imaging microscopy (FLIM), both upon chlorophyll *a* and phycobilisome (PBS) excitation. Fluorescence kinetics of single cells obtained using FLIM were compared with those of ensembles of cells obtained with time-resolved fluorescence spectroscopy. The global distribution of PSI and PSII and PBSs was mapped making use of their fluorescence kinetics. Both radial and lateral heterogeneity were found in the distribution of the photosystems. State transitions were studied at the level of single cells. FLIM results show that PSII quenching occurs in all cells, irrespective of their state (I or II). In *S. elongatus* cells, this quenching is enhanced in State II. Furthermore, the decrease of PSII fluorescence in State II was homogeneous throughout the cells, despite the inhomogeneous PSI/PSII ratio. Finally, some disconnected PBSs were resolved in most State II cells. Taken together our data show that PSI is enriched in the inner thylakoid, while state transitions occur homogeneously throughout the cell.

## Teaser

During state transitions, the ratio of quenched and unquenched photosystem II complexes is homogeneously changed in individual cells of the cyanobacterium *Synechococcus elongatus*.

## Introduction

Virtually all life in our biosphere is sustained by sunlight, which is harvested and transformed into chemical energy by the process of photosynthesis. In oxygenic photosynthesis, Photosystem I (PSI) and Photosystem II (PSII) are embedded in the thylakoid membrane and work in series to drive the electron transport from water to NAD(P)^+^. Photosynthetic organisms are subject to various types of light stress due to ever-changing illumination conditions (in terms of duration, intensity, and spectral composition). In response to light stress, photosynthetic organisms have developed strategies to avoid photodamage and/or to maintain the optimal rate of photochemistry. State transitions form a low-light acclimation response by which plants, algae, and cyanobacteria balance the excitation of PSI and PSII. The light that preferentially excites PSI leads to oxidation of the plastoquinone pool and drives the photosynthetic system to State I (Mullineaux and Allen, 1990; Mullineaux, 2014). The light that preferentially excites PSII reduces the plastoquinone pool and drives the photosynthetic system to State II (Mullineaux and Allen, 1990; Mullineaux, 2014).

In green plants, state transitions occur via the redistribution of light-harvesting complexes II (LHCII) between PSI and PSII (Allen et al., 1981; Staehelin and Arntzen, 1983; Wientjes et al., 2013; Rochaix, 2014). In State I, LHCII is closely associated with PSII and transfers most of the absorbed energy to PSII (Allen et al., 1981; Rochaix, 2014). In State II, the reduction of the plastoquinone pool results in phosphorylation and decoupling of a proportion of LHCII from PSII and its association with PSI (Allen et al., 1981; Rochaix, 2014). In cyanobacteria, phycobilisomes (PBSs) are considered to be the functional equivalents of LHCII in plants. Despite the fact that state transitions in cyanobacteria were already discovered in 1969 (Murata, 1969), a comprehensive understanding of the process is still lacking.

State transitions are characterized by a decrease in the fluorescence of PSII in State II (McConnell et al., 2002; Kirilovsky, 2015; Calzadilla et al., 2019). In cyanobacteria, this is observed when probed with light that is selectively absorbed by either PBSs or chlorophyll (Chl) *a* (Chukhutsina et al., 2015; Ranjbar Choubeh et al., 2018; Bhatti et al., 2020). A strong decrease of PSII fluorescence in State II observed upon Chl *a* excitation cannot be explained by the redistribution of PBSs between PSII and PSI (Mullineaux et al., 1997; McConnell et al., 2002). This immediately distinguishes the process of state transitions in cyanobacteria from that in plants. Generally, the proposed mechanism for state transitions in cyanobacteria has been conceptually similar to the mechanism in plants and green algae. An increase of the excitation-energy transfer (EET) to PSI at the expense of PSII in State II was suggested to occur in plants and green algae (Allen et al., 1981; Staehelin and Arntzen, 1983; Wientjes et al., 2013; Rochaix, 2014). In cyanobacteria, the proposed reduced EET to PSII reaction centers in State II was explained by different hypotheses: (1) (Re)Distribution of PBSs between PSII and PSI in *Synechococcus* 6301 was concluded to regulate the PBS-absorbed excitation energy (Allen et al., 1985); (2) The fluorescence decrease in State II in the cyanobacteria *Anacystis nidulans* and *Synechocystis* PCC 6803 was ascribed to the direct spillover of excitation energy from PSII to PSI (Bruce et al., 1985; Olive et al., 1997); (3) It was proposed by McConnell et al. (2002) that in a megacomplex of PBS–PSI–PSII, a decreased distance in photosystems in State II would result both in increased transfer of excitation energy from PBS to PSI and spillover of excitation energy from PSII to PSI in *Synechococcus* 7002 and *Synechocystis* 6803. However, in none of the abovementioned studies, the expected increase of PSI fluorescence was observed in State II at the expense of PSII fluorescence. By contrast, in plants, an increase in the PSI fluorescence at the expense of PSII fluorescence in State II is clearly observed using time-resolved fluorescence spectroscopy (Bos et al., 2019). This casts doubt whether a redistribution of excitation energy between PSI and PSII occurs during state transitions in cyanobacteria. In Ranjbar Choubeh et al. (2018), state transitions in *Synechococcus elongatus* were induced at RT and by rapidly freezing, the various states were “captured/stabilized” and studied at 77K. No evidence was found for the migration of PBSs from PSII to PSI in State II or for the spillover of energy from PSII to PSI. Instead, the observed decrease in PSII fluorescence in State II was ascribed to direct quenching of PSII complexes. However, it is still not clear if reversible physical changes in the association of PBSs and PSII are involved in the process, which leads to quenching of PSII in State II. The role of PBSs in state transitions is particularly ambiguous, since in *Synechocystis* 6803, *Synechococcus* 7002, and *Synechococcus* 7120, the mutants lacking a terminal emitter protein named ApcD show no fluorescence changes upon PBS excitation and a reduced PSII fluorescence quenching upon Chl *a* excitation in State II (Ashby and Mullineaux, 1999; Dong and Zhao, 2008; Dong et al., 2009; Calzadilla et al., 2019; Bhatti et al., 2020). In our recent work (Bhatti et al., 2020), it was indeed shown that in *Synechocystis* 6803 the PSII fluorescence decrease in State II observed upon photosystems excitation (Chl *a*) was only ∼9% in the *Synechocystis* ΔApcD as compared to 21% for WT cells. This suggests that PBSs are somehow involved in the process of state transitions in cyanobacteria.

Time-resolved fluorescence spectroscopy can in principle distinguish between the different scenarios discussed above to explain the decrease in the PSII fluorescence in State II. However, this approach usually provides an average over a very large ensemble of cells. Thus, the information about possible inhomogeneities in the spatial distribution of photosynthetic complexes in cyanobacteria cells is inaccessible with this method. In recent years, microscopy studies using either hyperspectral imaging (Vermaas et al., 2008) or labeling with fluorescence proteins (Casella et al., 2017; Konert et al., 2019) have shown that PSI, PSII, and PBSs are inhomogeneously distributed over the thylakoid membranes of cyanobacteria. In a fluorescence lifetime imaging microscopy (FLIM) study on *Synechocystis* 6803 cells, disconnected PBSs were observed in a fraction of the cells and large variations regarding the disconnection of PBSs were found between cells (Krumova et al., 2010). In line with these results, it appears very purposeful to study state transitions by mapping the fluorescence kinetics at the subcellular level in living cyanobacteria, as our understanding of these processes in cyanobacteria remains poor.

In the present work, we used confocal FLIM to study state transitions in *S. elongatus* cells at room temperature. These cells have a cylindrical shape, with typical dimensions of ∼2.5 µm × 1.1 µm × 1.1 µm (Moronta-Barrios et al., 2013). The thylakoid membrane is located near the periphery of the cell and consists typically of four membrane layers, which are connected and enclose a single lumen (Nevo et al., 2007). To investigate the spatial distribution of PBSs, PSI, and PSII in the thylakoid membrane and their role in state transitions, selective excitation of PBSs and Chl *a* was used. The results were compared with ensemble time-resolved fluorescence measurements, which we reported earlier (Bhatti et al., 2020).

## Results

### Fluorescence lifetime imaging of *S. elongatus*

The aim of this work is to refine the understanding of the state-transitions process in cyanobacteria by investigating the role of PSI, PSII, and PBSs at the cellular level using FLIM. For this purpose, the cells were probed in States I and II after selective excitation of photosystems (440-nm excitation) and PBSs (577-nm excitation). All measurements were performed at room temperature. State I was induced by keeping the cells for 30 min in blue light. State II was induced by keeping the cells in darkness. The same cells were, respectively, imaged for States I and II. The State I cells were also illuminated with blue light during the measurement time of 60 s. The fluorescence kinetics at the cellular level obtained in this work are compared with the fluorescence kinetics of an ensemble of *S. elongatus* cells reported earlier (Bhatti et al., 2020).

### Experimental conditions for FLIM

Direct excitation of the photosystems at 440 nm allowed us to image the fluorescence kinetics of PSI and PSII from *S. elongatus* cells in States I and II. The 577-nm excitation was used to probe the interaction of PBSs with photosystems in different states. The time-resolved fluorescence was recorded at 685–720 nm, where both PSI and PSII have significant emissions.

Our previous time-resolved spectroscopic studies at room temperature and 77K have revealed that state transitions in cyanobacteria are characterized by a reversible decrease in the average PSII fluorescence lifetime in State II (Chukhutsina et al., 2015; Ranjbar Choubeh et al., 2018; Bhatti et al., 2020). However, the fluorescence kinetics of PSII is also influenced by the laser light intensity. At very low excitation intensities, due to photochemical quenching of excitations by open PSII reaction centers, PSII fluorescence shows faster kinetics (Tian et al., 2013). At relatively high light intensities, the PSII fluorescence kinetics becomes slower due to the closure of PSII reaction centers (Tian et al., 2013). The PSI fluorescence kinetics are equally fast (∼20 ps) for open and closed RCs, and they are thus independent of the light intensity (Gobets et al., 2001). In this work, all measurements were done with closed PSII reaction centers to completely separate the PSI and PSII fluorescence kinetics from each other. In laser scanning confocal microscopy, a diffraction-limited laser spot scans the sample to obtain a fluorescence-based image. An accurate determination of the excitation scanning parameters is essential to reliably image the state transitions in individual cells of cyanobacteria. Whereas the laser intensity should be high enough to close the PSII reaction centers, it should also be low enough to avoid singlet-singlet and singlet-triplet annihilation, which give rise to additional decay pathways. Furthermore, the dose of laser light that the cells receive should be limited to avoid photodamage, which would lead to the creation of quenchers and as such would shorten the excited-state lifetime (Nozue et al., 2016). The control experiments performed to find the correct illumination conditions are described in Supplemental Figures S1 and S2 for 440- and 577-nm excitation, respectively. Based on these measurements, 440- and 577-nm laser light intensities were selected that close the PSII RCs, do not lead to photodamage during the measurement time, and allow for reversible state transitions.

### Chl *a* excitation

**Figure 1:**
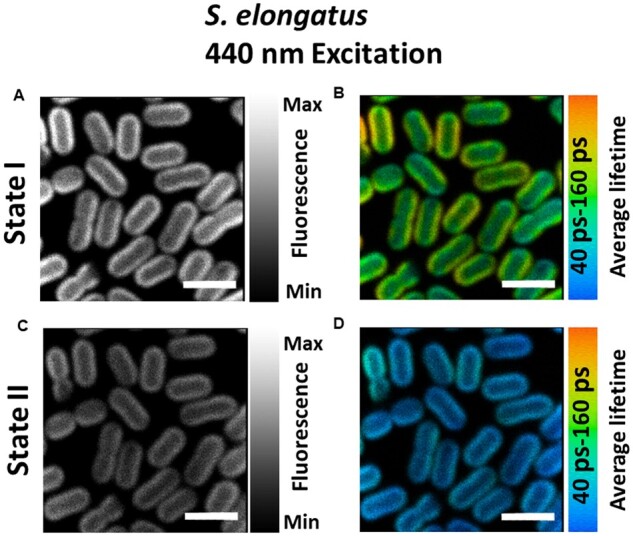
Confocal fluorescence microscopy images of *S. elongatus* cells adapted to States I and II. A and C, Steady-state intensity images of cells in States I and II, respectively. B and D, Average lifetime distributions in States I- and II-adapted cells. Same cells were imaged for both States I and II measurements. All images are equally adjusted for brightness and contrast. Excitation wavelength was 440 nm and fluorescence was recorded at 685–720 nm. Measurements were done at room temperature. Scale bar is 4 µm. See Supplemental Figure S7 for reproducibility of States I and II observed upon selective excitation of Chl *a*.

Fluorescence intensity and fluorescence lifetime images upon 440-nm excitation were recorded for the same *S. elongatus* cells first brought to State I (30 min blue light) and next to State II (30 min darkness). As the thylakoid membrane of *S. elongatus* cells is aligned with the cell wall, most of the fluorescence was measured at the periphery of the cells, whereas the inner regions of the cells showed very little fluorescence. State I cells (Figure 1A) showed significantly higher fluorescence as compared to State II cells (Figure 1C). Fluorescence decays were recorded using the time-correlated single-photon counting (TCSPC) method for each pixel and three lifetimes ($\tau_{1}$= ∼20–25 ps, $\tau_{2}$= ∼170–180 ps, and $\tau_{3}$= ∼700–900 ps) were sufficient to fit the fluorescence decay across the whole image. Values of these lifetimes were in good agreement with those determined from the measurements on the bulk of cells using a streak camera (Supplemental Figure S3). The short lifetime component ($\tau_{1}$= ∼20–25 ps) corresponds to the kinetics of PSI excitation trapping (Supplemental Figure S3; Gobets et al., 2001; Bhatti et al., 2020). The other two lifetimes ($\tau_{2}$= ∼170–180 ps and $\tau_{3}$= ∼700–900 ps) represent the biphasic fluorescence decay of PSII with closed reaction centers (Supplemental Figure S3; Tian et al., 2013; Bhatti et al., 2020). Figure 1B and D shows the distribution of the average lifetime for cells in States I and II, respectively. Cells in State II show a shorter average fluorescence lifetime (Figure 1D) than cells in State I (Figure 1B), which is consistent with the lower amount of steady-state fluorescence from State II cells in Figure 1C.

**Figure 2:**
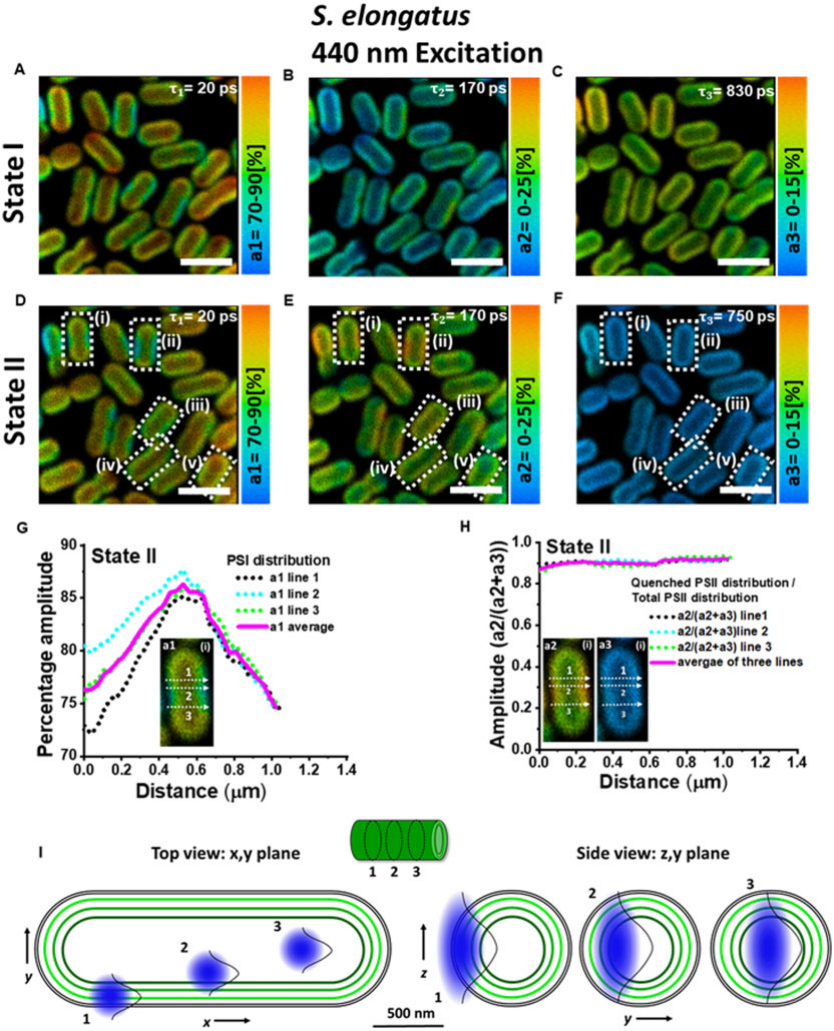
Distribution of amplitudes associated with the fluorescence lifetimes in *S. elongatus* cells in States I and II. A–C, State I. D–F, State II. Corresponding lifetimes are written in the figures. Excitation wavelength was 440 nm and fluorescence was detected at 685–720 nm. Images are adjusted for brightness and contrast. Measurement was done at room temperature. Scale bar is 4 µm. On five representative cells (i–v) in State II (lower panel) enclosed in white rectangles, further analysis for State II characterization was performed. G, The profile of amplitude (a1) across three lines along the short axis of the first cell “(i).” H, The distribution of a2/(a2+a3) across the same three lines as in (G). Cell “i” is shown in inset in (G) and (H). Analysis on cells “ii–v” is shown in Supplemental Figure S5. I, Schematic representation of the dimensions of the thylakoids (green) in *S. elongatus* with respect to the confocal observation volume (blue). Three observation volumes are indicated in the *x, y* and *y, z* plane.

The amplitude of each lifetime components reflects the relative contribution of the component to the total fluorescence decay. Amplitudes associated with the obtained lifetimes are shown in Figure 2A–C for cells in State I and in Figure 2D–F for cells in State II. The ∼20-ps component, representing the fluorescence kinetics of PSI, has the largest amplitude and contributes ∼80% to the total decay in both states (Figure 2A and D).

The amplitude of the ∼170-ps component, originating from PSII, has a much higher magnitude in State II cells (Figure 2E) as compared to State I cells (Figure 2B). This observation is in line with the increase in the amplitude of ∼180-ps component ($\lambda_{\max}=683\;nm$) observed using the streak camera, as cells transition from State I to State II (Supplemental Figure S3). The opposite effect is seen for the amplitude of the ∼750–830-ps component of PSII which is lower in State II-adapted cells. Higher/lower amplitude of the slow component in State I/II in Figure 2C and F corresponds well with the amplitude changes of the ∼700- and 1,000-ps component ($\lambda_{\max}=683\;nm$) observed with streak camera-ensemble measurements on *S. elongatus* cells (Supplemental Figure S3). Therefore, it is concluded that the higher amplitude of the 170-ps component in Figure 2E as compared with Figure 2B stems from the proportion of PSII complexes that become quenched in State II, resulting in shortening of their excited-state lifetime. Due to the smaller population of unquenched PSII complexes in State II, the amplitude of the slowest component in State II (Figure 2F) is smaller as compared to State I (Figure 2C).

Characterization of State II using FLIM can reveal if there is a relation between the PSI/PSII ratio and quenching of PSII. In five cells (i–v) in State II, enclosed in white rectangles in Figure 2D–F, a profile of PSI distribution and PSII quenching was acquired across the width of the cells (Figure 2G and H) and Supplemental Figure S4. The profiles of PSI distribution and PSII quenching were acquired in the same way as the thylakoids fluorescence was mapped across the short axis of the *Synechococcus* cells in Mahbub et al. (2020). Figure 2G shows an increase in the percentage distribution of PSI from the periphery toward the center of the cell across three lines along the short axis of one such representative cell. However, Figure 2H shows a nearly homogeneous distribution of quenched PSII/total PSII along the same three lines as in Figure 2G. Thus the comparison of Figure 2G and H shows that the increased quenching of PSII in State II is independent of the PSI distribution (see Supplemental Figure S4 for the analysis of 4 different cells). It must be noted that the central region of the cyanobacteria is free of thylakoids and therefore no fluorescence is emitted from this region. However, cyanobacterial cells are cylindrical in shape and the thylakoid membranes follow this shape in the cell (Figure 2I). Due to the poor axial resolution of the objective lens (Δz ∼0.6 µm), fluorescence will be observed from the inner thylakoids that are present above and below the focal plane of the focal volume. This fluorescence then appears to originate from the center of the cells as depicted in Figure 2I. This explains the highest amplitude of the a1 component (representing PSI) in the middle of the cells in Figure 2G and Supplemental Figure S4. Supplemental Figure S5 (showing analysis at a lower pixel binning) shows that the high amplitude of the a1 component in the middle of the cells is not an artifact of the binning. The schematic in Figure 2I illustrates that the diffraction-limited lateral and axial resolutions of the microscope combined with the pixel binning greatly reduces the observation of PSI heterogeneity between the inner and peripheral thylakoid layers, which are spaced by ∼80 nm. This means that the modest difference in a1, of 5–10%, that we observed between the outside and the inside of the cell should be caused by a far larger heterogeneity in the PSI distribution.

**Figure 3:**
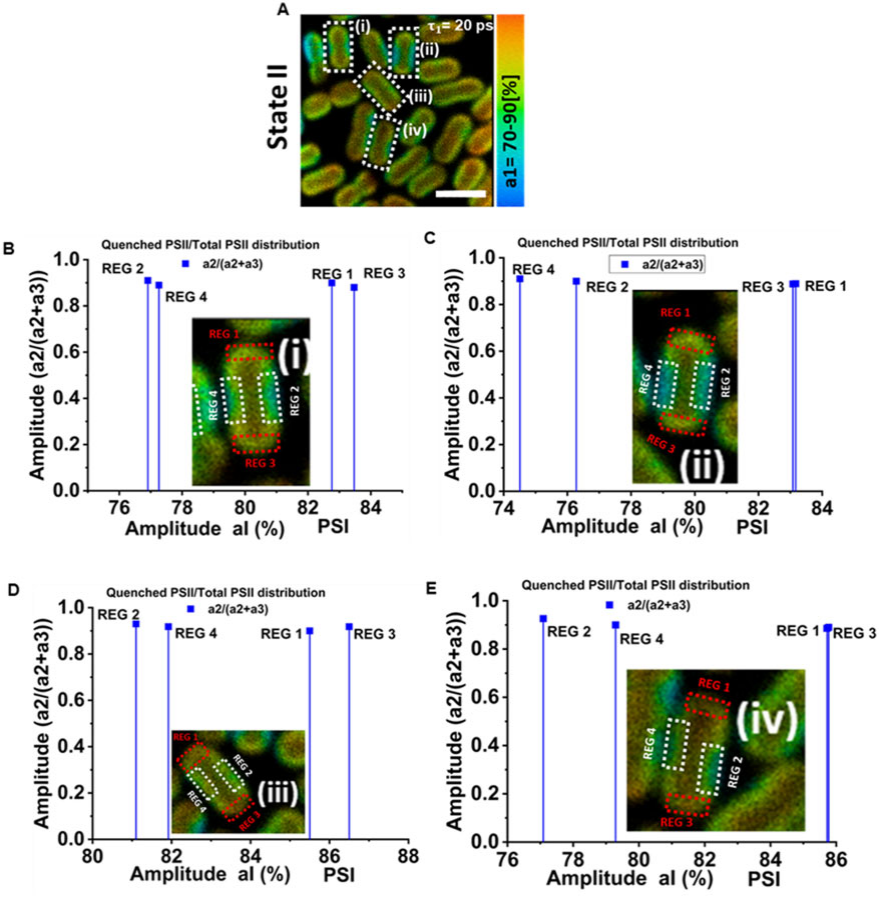
Correlation between PSI distribution and PSII quenching. A, Distribution of amplitude associated with PSI fluorescence lifetime component (20 ps) in State II cells. On cells “i–iv” marked within white rectangles, further analysis was performed to investigate the correlation between the PSI percentage and ratio of quenched PSII/total PSII (a2/[a2+a3]) in specific cell regions. B–E, Fraction of quenched PSII of (a2/[a2+a3]) versus the average PSI amplitude in REG 1, REG 2, REG 3, and REG 4 of the cells “i–iv”, respectively. Cells “i–iv” are shown in inset. “REG 1” and “REG 3” shown with red rectangles represent domains in the cell with higher PSI content. “REG 2” and “REG 4” shown with white rectangles represent domains in the cell with lower PSI content.

Furthermore, in most of the cells, there were clearly identifiable regions that showed lower or higher amplitude of the 20-ps (PSI) lifetime component, indicating a lower or higher PSI/PSII ratio. In Figure 3A, in four cells (i–iv) in State II (enclosed in white rectangles) a comparison of PSII quenching (a2/[a2+a3]) was made between the regions of high PSI concentration and low PSI concentration. In Figure 3B–E, cells “i–iv” are shown with respective regions of high PSI content (REG 1 and REG 3) marked with red rectangles and regions of low PSI content (REG 2 and REG 4) marked with white rectangles. Figure 3B–E shows that the quenching of PSII in State II is similar in the regions of low PSI/PSII ratio and in those of high PSI/PSII ratio, in agreement with the results in Figure 2G and H.

Table 1 shows the comparison of lifetimes and amplitudes obtained from the analysis of FLIM and streak camera data in the 685–720 nm range. The fluorescence decrease in State II as observed in the FLIM measurements was found to be 39.4 ± 5.1% (for three biological replicates) which is comparable with the 39.6 ± 1.1% decrease observed in streak camera measurements. The large similarity of the measured values shows that there is a good agreement between the amplitudes resolved with FLIM and streak.

**Table 1:** Comparison of lifetime components and lifetime-associated amplitudes obtained from streak camera measurements and FLIM measurements

	Lifetimes		Amplitude	State I (%)	State II
Streak camera	τ_1_	∼20–25 ps	A1(τ_1_)	80.1 ± 1.40	79.9 ± 1.2
	τ_2_	∼180–200 ps	A2(τ_2_)	9.7 ± 0.6	16.2 ± 1.1
	τ_3_	∼0.8–1.0 ns	A3(τ_3_)	10 ± 0.8	3.7 ± 0.8
FLIM	τ_1_	∼20 ps	A1(τ_1_)	83.3 ± 0.7	81.9 ± 1.9
	τ_2_	∼170–200 ps	A2(τ_2_)	8.75 ± 1.3	15.2 ± 1.8
	τ_3_	∼0.75–0.9 ns	A3(τ_3_)	8.9 ± 1.5	3.4 ± 1.0

The 430 nm and 440 nm excitations were used for streak camera measurements and FLIM measurements, respectively. For the streak camera results, amplitudes A1 ($\tau_{1})$, A2 ($\tau_{2})$, and A3 ($\tau_{3})$ represent the average percentage contribution of individual amplitudes to the total fluorescence in the 685–720 nm fluorescence detection range. For the FLIM results, amplitudes A1 ($\tau_{1})$, A2 ($\tau_{2})$, and A3 ($\tau_{3})$ represent the average of the percentage distributions of a1, a2, and a3, respectively. Average values and SDs for amplitudes are for three biological replicates.

### PBS excitation

**Figure 4:**
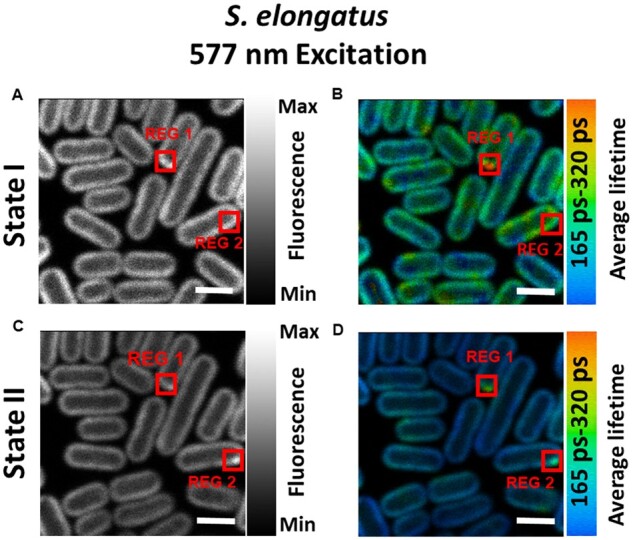
Confocal fluorescence microscope images of *S. elongatus* cells adapted to States I and II. A and C, Steady-state intensity images of cells in States I and II, respectively. B and D, Average lifetime distributions in States I- and II-adapted cells. Same cells were imaged for both States I and II measurements. Excitation wavelength was 577 nm and fluorescence was recorded at 685–720 nm. Measurements were done at room temperature. “REG 1” in all figures, enclosed in red boxes, shows the area where fluorescence (A and C) and average lifetime (B and D) is higher than the rest of the cell. “REG 2” shows the area of high fluorescence (C) and average lifetime (D) in State II cells. “REG 2” in (A and B) is drawn for comparison with “REG 2” in (C and D). All images are equally adjusted for brightness and contrast. Scale bar is 2 µm. See Supplemental Figure S8 for reproducibility of States I and II observed upon selective excitation of PBSs.

In cyanobacteria, PBSs increase the light absorption and are the major antennas of PSII (Watanabe et al., 2014). The role of PBSs in state transitions is largely ambiguous so far. To selectively excite the PBSs, 577-nm excitation pulses were used for the lifetime imaging of state transitions in *S. elongatus* cells. The steady-state fluorescence intensity and average fluorescence lifetime of cells in State I (Figure 4A and B) are both higher than those of State II cells (Figure 4C and D), similar to the case of Chl *a* excitation.

Upon PBS excitation, in a small fraction of cells, some regions of high fluorescence were observed (see Figure 4A (State I) and 4C (State II) “REG 1”). Figure 4B and D show that the average lifetime in this region also has a higher value. The fluorescence of PBSs, which are connected to the photosystems, decays with a lifetime of 120 ± 20 ps (Krumova et al., 2010). However, fluorescence decay kinetics of PBSs becomes slower in the case of free and/or partially connected PBSs. Therefore, a higher value of the average lifetime and fluorescence in some regions of a few cells, observed only after PBS excitation, is ascribed to free and/or partially connected PBSs. In State II, both fluorescence and average lifetime in “REG 1” were reduced as compared to State I, indicating that PSII is also present in this region. In State II, another region of high fluorescence and average lifetime was identified in another cell, which was designated as “REG 2”.

**Figure 5:**
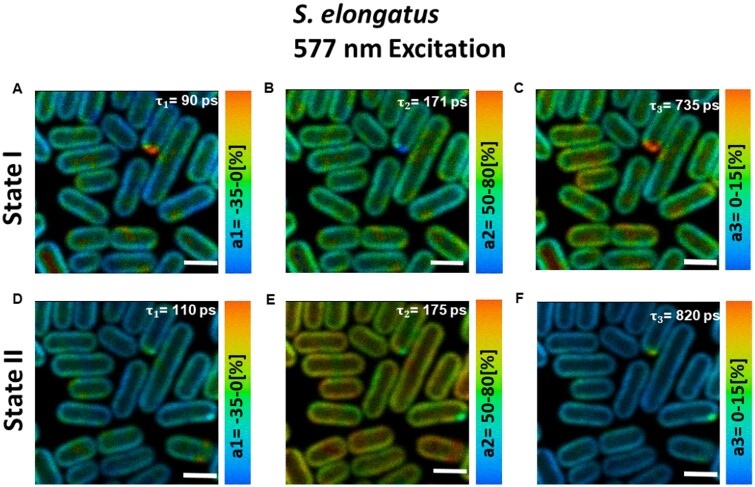
Distribution of amplitudes associated with the fluorescence lifetimes in *S. elongatus* cells in States I and II. A–C, State I. D–F, State II. Corresponding lifetimes are written in the figures. Images are adjusted for brightness and contrast. Excitation wavelength was 577 nm and fluorescence was detected at 685–720 nm. Measurements were performed at room temperature. Scale bar is 2 µm.

In the detection range of 685–720 nm, three-lifetime components ($\tau_{1}$= ∼90–110 ps, $\tau_{2}$= ∼170–190 ps, and $\tau_{3}$= ∼700–1,000 ps) were found sufficient to fit the fluorescence decay across the whole image. These lifetimes are in very good agreement with the ones obtained from the streak camera measurements on the bulk of *S. elongatus* cells (Supplemental Figure S6). Figure 5 presents the distribution of the fluorescence amplitudes associated with the obtained lifetime components for cells in State I (Figure 5A–C) and State II (Figure 5D–F), respectively. The 90–110-ps component with mostly negative amplitude was very similar for cells in State I (Figure 5A) and State II (Figure 5D) and corresponded well with the ∼90-ps component in Supplemental Figure S6 streak camera measurements. This component has been reported to represent EET within PBSs and from PBSs to photosystems (Bhatti et al., 2020). The ∼170-ps decay component had the highest amplitude in both states and corresponded with the ∼180-ps component ($\lambda_{\max}=683\;nm$) in streak camera measurements in Supplemental Figure S6. In State II, this component had a higher amplitude (Figure 5E) than in State I (Figure 5B) across the whole cell and corresponded with the ∼180-ps component in Bhatti et al. (2020). Based on our previous work (Bhatti et al., 2020), the ∼170-ps component is ascribed to quenched PSII.

**Figure 6:**
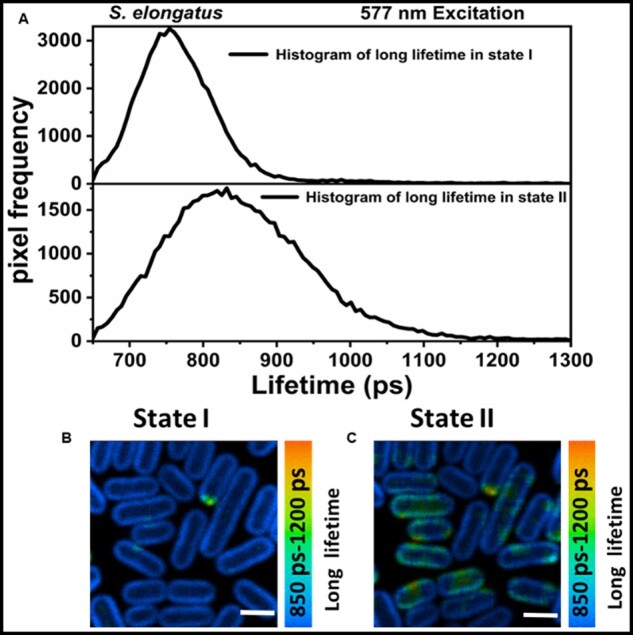
Distribution of long lifetime at 577 nm excitation. A, The histogram of long lifetime in State I (upper panel) and State II (lower panel), respectively. Distribution of the long lifetime in range of 850–1,200 ps in State I (B) and State II (C). (B) and (C) are corrected for the brightness and contrast. Distribution of the slow lifetime was obtained by fixing the first two lifetimes in the analysis, whereas the third lifetime was left free. Scale bar in (B) and (C) is 2 µm.

The third lifetime component ($\tau_{3}$= ∼735 ps [State I], 820 ps [State II]) in Figure 5 had considerably higher amplitude in State I as compared to State II and was comparable with the ∼0.8–1.0-ns component ($\lambda_{\max}=683\;nm$) in streak measurements in Supplemental Figure S6 representing slow PSII decay and decay from some disconnected PBSs. The lifetime of this component reproducibly had a slightly higher value in State II as compared to State I in our FLIM measurements. This observation is in agreement with the previous time-resolved spectroscopic works on *S. elongatus* (Bhatti et al., 2020) and *Synechococcus* 6301 (Mullineaux et al., 1990). Interestingly, this was not the case upon Chl *a* excitation. The histogram of the long lifetime (Figure 6A) shows that for State II cells, the amount of pixels with longer lifetimes (850–1,200 ps) is higher than for State I cells. The distribution of the long lifetime in the range of 850–1,200 ps for cells in States I and II is shown in Figure 6B and C, respectively.

## Discussion

State transitions in cyanobacteria have been extensively studied for many decades; however, no consensus has been reached so far about the exact roles of PSI, PSII, and PBSs during the process. In this work, we apply FLIM to investigate state transitions at the cellular level in living *S. elongatus* cells. FLIM was used to visualize the distribution of PSI, PSII, and PBSs based on their autofluorescence. As such, the need to introduce fluorescent tags on photosynthetic proteins was avoided and state transitions could be followed under near-native conditions. The fluorescence kinetics of cells in State I were compared with the same cells in State II. The FLIM results were also compared with the streak camera data to better interpret the fluorescence kinetics obtained from the cell ensembles. The main objective of this work was to bring clarity about the roles of PSI, PSII, and PBSs by correlating the distribution of fluorescence changes during state transitions with the distribution of photosynthetic pigments in thylakoid membranes.

### Distribution of photosystems and state transitions

Upon selective excitation of the photosystems (440-nm excitation), a ∼20-ps fluorescence component from PSI and two lifetimes components of ∼180 ps and ∼750–820 ps from PSII (Figure 2) were resolved. FLIM results (Figure 2) show that heterogeneity of the PSI and PSII distribution in *S. elongatus* cells occurs in two ways. Firstly, PSI appears to be more abundant in the inner thylakoid(s) as indicated by the gradual increase in the percentage amplitude of the PSI representative fluorescence signal (∼20 ps component) from periphery toward the inner regions of the five representative cells (Figure 2;  Supplemental Figure S4). The percentage distribution of PSII decreases from outer thylakoid(s) to inner thylakoids. The enrichment of PSI in the inner thylakoid layers has been reported before for *Synechococcus* PCC 7942 (Casella et al., 2017) and *Synechocystis* 6803 (Vermaas et al., 2008; Collins et al., 2012). Additionally, pockets of high PSI (low PSII) content and low PSI (high PSII) content (Figure 3) were found in both states in the thylakoids of individual cells. Despite ∼30 min of difference in State I and II measurements, the location of regions of high/low PSI/PSII ratio was the same in both states. This type of lateral and partial segregation of PSI and PSII, composing a mosaic-like structure of stable microdomains in thylakoids of *Synechocystis* sp. PCC 6803, was recently reported (Strašková et al., 2019). This microscale heterogeneous organization of PSI and PSII in cyanobacterial thylakoids was speculated to be an evolutionary/functional precursor for the granal/stromal heterogeneity in photosystems in higher plants (Konert et al., 2019; Strašková et al., 2019). Clusters enriched in PSI and PSII in thylakoids of *S. elongatus* were also visualized (Casella et al., 2017)*.* In various species of cyanobacteria, a lateral segregation of PSI in thylakoid membranes was resolved (MacGregor-Chatwin et al., 2017). However, a complete separation of PSI and PSII has not been observed in this study or the previous studies (Strašková et al., 2019).

Upon State I to State II transition, a decrease in the amplitude of the slowest component (735–820 ps) was observed with a concomitant increase in the amplitude of the ∼180-ps component as compared to State I (Figure 2), similar to the streak camera measurements on an ensemble of cells. As a result, the average lifetime and fluorescence intensity for State II was reduced compared to cells in State I (Figure 1). The reduced PSII fluorescence is nearly homogeneous (Figures 2 and 3; Supplemental Figure S4) in all parts of the cells, irrespective of the inhomogeneous distribution of PSI and PSII throughout the cell. This observation shows that the amount of PSI does not correlate with the level of State II transition in *S. elongatus*.

### PSII quenching in States I and II

In this work, upon selective Chl *a* excitation, a component of 170–190 ps was observed in both states. Recent studies have shown that this component represents fluorescence decay of PSII (Tian et al., 2013; Bhatti et al., 2020). An increase in the amplitude of this component in State II at the expense of the slowest component (Figure 2) was concluded to show the quenching of PSII in State II (Bhatti et al., 2020). However, as this decay lifetime is present in both states, it can be suggested that also in State I the quenching of PSII complexes exists already for part of the complexes. Based on these observations, we propose that in *S. elongatus* a certain quenching process intrinsic to the PSII core exists independent of State I or II. Only the extent of PSII quenching is increased in State II to balance the photochemistry of PSI and PSII. Furthermore, very similar fluorescence kinetics observed at room temperature for States I and II in *S. elongatus*, *Synechocystis* 6803 (Bhatti et al., 2020), and *Synechococcus* 6301 (Mullineaux et al., 1990) suggest that the state transition process is similar in different cyanobacteria species.

### The 577-nm excitation: fluorescence quenching and disconnection of PBS

The 577-nm excitation was used to selectively excite the PBSs in FLIM measurements and fluorescence was detected at 685–720 nm. Based on the fluorescence kinetics, the distribution of PBSs and PSII was imaged across the cells. As was found for Chl *a* excitation, a significant decrease in the average lifetime and fluorescence intensity was observed in State II as compared to State I (Figure 4), which is ascribed to PSII quenching in State II in agreement with earlier results (Bhatti et al., 2020).

The FLIM results show that in some cells, there are regions where the average lifetime is significantly longer than in the rest of the cell. We ascribe this to the contribution of disconnected PBSs, which have a lifetime of 1.4–1.6 ns (Tian et al., 2012). Figure 4B shows one such region “REG 1” in State I cells. In State II, an additional region, designated as “REG 2,” was identified (Figure 4D). At least two clearly identifiable regions of disconnected PBSs in State II (Figure 4D) as compared to only one prominent region in State I (Figure 4B) show that the disconnection of the PBSs can occur during the time of the experiment. It is important to note that with FLIM, both the presence and the location of disconnected PBSs can be resolved, which is not the case for ensemble measurements. However, it should be noted that such type of disconnection of PBSs was only observed in some of the replicates.

After 577-nm excitation, the longest lifetime in State II cells represents the average of unquenched PSII and slowly decaying disconnected/poorly connected PBSs. The comparison of the distribution of the longest lifetime component in the 850–1,200 ps range in State I (Figure 6B) and State II (Figure 6C) indicates that regions of disconnected/poorly connected PBSs are present in a majority of State II cells. Fluorescence from such regions in State II slightly offsets the fluorescence decrease observed at 683 nm in State II, due to the quenching of PSII. PBSs are water-soluble proteins and their mobility along the thylakoid membranes of state-transition performing cyanobacterium *S. elongatus* (Joshua and Mullineaux, 2004) and mesophilic red algae Porphyridium* cruentum* (Kaňa et al., 2014) has been shown with FRAP microscopy. A slow decoupling of PBSs in *Synechocystis* sp. PCC6803 after a long-term exposure (>1 h) to high light was observed with confocal microscopy (Steinbach and Kaňa, 2016). High intensities of light, absorbed either by PBSs or photosystems, induce an excitonic decoupling of PBSs followed by their disassembly and detachment from thylakoid membrane (Tamary et al., 2012). This type of uncoupling of PBSs occurs at the timescale of a few seconds and the physiological purpose is the photoprotection of reaction-center proteins (Tamary et al., 2012). A very small fraction of uncoupled PBSs resolved in this work and previously (Krumova et al., 2010) at very low excitation powers does not appear to have any physiological significance for photoprotection or state transitions. It cannot, however, completely be ruled out that the laser power used to close the reaction centers also induces some uncoupling of a very small amount of PBSs. The higher value of the longest lifetime in State II is most probably caused by the presence of a fraction of disconnected PBSs in both states. In State II, the relative contribution of PSII to the longest lifetime is smaller and therefore the average value of the longest lifetime will be influenced more by the long lifetime of the disconnected PBSs

### PBSs and PSII quenching in State II

In this work, we have shown that increased PSII quenching in State II as compared to State I occurs for state transitions in cyanobacteria, and that this is responsible for the commonly observed change in the ratio of PSII/PSI fluorescence. As PBSs do not play a role in redistributing the energy between PSI and PSII (Ranjbar Choubeh et al., 2018), it is tempting to conclude they are not involved at all in state transitions. However, upon selective excitation of PSs, the ΔApcD mutant strains of *Synechocystis* 6803 (Calzadilla et al., 2019; Bhatti et al., 2020) and *Synechococcus* 7002 (McConnell et al., 2002) show reduced PSII quenching in State II as compared to their wild-type counterparts. The ApcD component is situated at the bottom of the PBS core and is in direct interaction with the PS complexes (Zhang et al., 2017). It thus appears that the interaction between PBS and PS plays a role in state transitions, possibly by regulating the reorganization of PSII complexes. The exact mechanism by which PBSs are involved in state transitions remains to be investigated.

## Conclusions

This study shows that in *S. elongates*, two (sub)populations of PSII, namely quenched and unquenched, exist in States I and State II. The equilibrium between quenched and unquenched PSII is changed upon state transitions. The PSII fluorescence quenching in State II was found to be nearly homogeneous throughout the cells. The exact mechanism of PSII quenching in State II is yet to be determined. However, taking into consideration the observations from previous studies, it may be that the interaction between PBS and PSII needs to allow for a reorganization of PSII complexes in State II, which are required for PSII quenching.

## Materials and methods

### Sample preparation

*Synechococcus* PCC 7942 (*S. elongatus*) cells were grown in BG-11 medium (20 mL/L) supplemented with sodium bicarbonate (0.85 g/L) and sodium nitrate (1.75 g/L) at pH ∼8.0. Cells were grown at 30°C under white light illumination at 50 µmol photons m^−2^s^−1^ in 250-mL flasks shaken at 100 rpm, containing culture volumes of 60 mL as described previously (Ranjbar Choubeh et al., 2018). Cells in the logarithmic phase of growth at optical density ∼0.6–0.7 at 800 nm (${OD}_{800})$ for a 1-cm path length were harvested for the measurements. Harvested cells were then centrifuged at 1,500 *g* for 10 min and immobilized on agar-coated microscopic slides (∼2% [w/v] invitrogen select agar mixed with growth medium). Immediately prior to the measurements, microscopic slides were illuminated for 30 min with blue light (438 ± 22 nm at ∼40 µmol photons m^−2^s^−1^) to induce State I. Cells adapted to State I were kept under the same light condition for the duration of the measurement as described previously (Bhatti et al., 2020). The combined sample handling and measurement time were less than 5 min. Afterward, the same slides were kept in darkness for 30 min to induce State II and the same cells were measured.

### FLIM measurements

Measurements were performed on an (inverted) confocal Leica TCS SP8 Hyd fluorescence microscope. A 63 × 1.20 NA water immersion objective was used to image the cells. The confocal pinhole diameter of the microscope was 0.5–0.8 AU. The lateral and the axial resolution of the microscope were ∼0.2 µm and ∼0.6 µm, respectively. Chl *a* was excited at 440 nm using a diode pulsed laser and PBSs were excited at 577 nm using a pulsed supercontinuum laser. At 440 nm excitation, 0.025 µW laser power was used. At 577 nm excitation, 0.010 µW laser power was used. The laser repetition rate in both cases was 40 MHz and the scan rate was 400 Hz. Fluorescence was detected at 685–720 nm with internal hybrid detectors coupled to TCSPC boards (SPC-152, Becker & Hickl GmbH), as described previously (Nozue et al., 2016). Images were recorded for 60 s upon Chl *a* excitation and for 20 s upon PBS excitation. The total image size was either 48 µm × 48 µm or 29 µm × 29 µm with 256 × 256 pixels. The time step for the TCSPC detection was 12 ps/channel.

FLIM images were analyzed using image analysis software (SPCImage, Becker & Hickl GmbH). Pixel binning of 5 or higher (maximum 8) was used. A set of very reproducible lifetimes for different cells and different biological samples was found from the global analysis of FLIM images. Pinacyanol chloride dissolved in methanol with lifetime of 6 ps (van Oort et al., 2008) was used to measure the instrument response function for the FLIM measurements. All measurements were performed at room temperature.

## Supplemental data

**Supplemental Figure S1.** Fluorescence kinetics at 440-nm laser scanning.

**Supplemental Figure S2.** Fluorescence kinetics at 577-nm laser scanning.

**Supplemental Figure S3.** Time-resolved fluorescence spectroscopy on *S. elongatus* cells at 440-nm excitation.

**Supplemental Figure S4.** Profiles of PSI distribution and PSII quenching across the width of *S. elongatus* cells.

**Supplemental Figure S5.** FLIM analysis of *S. elongatus* cells at low pixel binning.

**Supplemental Figure S6.** Time-resolved fluorescence spectroscopy on *S. elongatus* cells at 577-nm excitation.

**Supplemental Figure S7.** Confocal fluorescence microscopy images of *S. elongatus* cells adapted to States I and II with excitation at 440 nm.

**Supplemental Figure S8.** Confocal fluorescence microscopy images of *S. elongatus* cells adapted to States I and II, with excitation at 577 nm.

## Supplementary Material

kiab063_Supplementary_Data

## Notes

A.F.B., H.v.A., and E.W. conceived the experiments; A.F.B. performed the experiments and analyzed the data. A.F.B. wrote the manuscript with contributions of all authors; D.K., H.v.A., and E.W. supervised the experiments and writing process. E.W. agrees to serve as the author responsible for contact and ensures communication.

The author responsible for distribution of materials integral to the findings presented in this article in accordance with the policy described in theInstructions for Authors (https://academic.oup.com/plphys/pages/general-instructions) is: Emilie Wientjes (emilie.wientjes@wur.nl).
