# Supplementary material for: State transitions and photosystems spatially resolved in individual cells of the cyanobacterium Synechococcus elongatus
Source: Plant Physiol. 2021 Feb 10;186(1):569–80. doi: 10.1093/plphys/kiab063 (PMC8154081; doi:10.1093/plphys/kiab063)
Supplement: kiab063_Supplementary_Data [file kiab063_supplementary_data.docx]

**Supplementary Information**

**
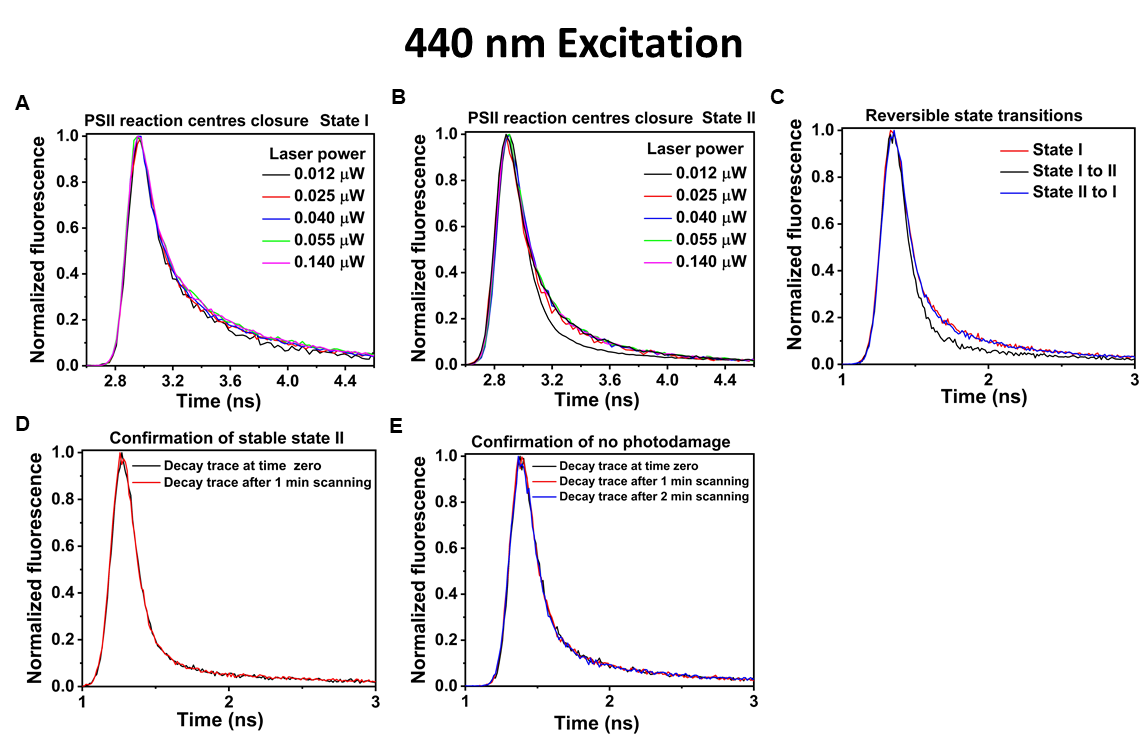
**

**Supplemental Figure S1.** **Fluorescence kinetics at 440 nm laser scanning.** Fluorescence kinetics as a function of power of excitation pulses for cells in state I (A) and state II (B). (C) Decay traces from cells equilibrated in state I (red solid line), cells brought from state I to state II (black line) and cells brought back to state I (blue line). (D) Black line shows a decay trace recorded for 1 sec after fully adapting the cells in state II and is indicated as time zero trace. Red line shows the decay trace at the end of 1 minute of laser scanning. (E) Black, red and, blue lines show the decay traces at the beginning, after 1 minute and after 2 min of laser scanning of state I adapted cells. 440 nm excitation pulses were used and fluorescence was detected at 685-720 nm. All measurements were performed at room temperature. Cells were adapted for 30 minutes to blue light to induce state I and were adapted to darkness for the same time to induce state II. Note that the measurements in A,B and C,D,E were performed with the difference of several days. The IRF of the TCSPC-microscopy was broad in case of measurements shown in A,B as compared with the measurements shown in C,D,E. For the correct estimation of the fluorescence decays the IRF for each experiment is measured as described in the materials and methods section. The length of the time axis in A-E is 2 ns.

In Fig. S1A fluorescence kinetics for excitation powers of ~0.01 µW to 0.14 µW (440 nm light) are relatively similar. For state II cells in Fig. S1B the PSII fluorescence kinetics is much faster at ~0.01 µW power as compared to the kinetics obtained after 0.025 µW of laser power excitation. However, the fluorescence kinetics did not slow down further when the power was increased even up to 0.14 µW. It shows that the PSII reaction centers were closed at all powers except at the lowest one (0.1 µW). In comparison in state I (Fig. S1A) PSII reaction centers are predominantly closed for all laser powers tested. All further measurements at 440 nm excitation were performed with 0.025 µW laser power. Fig. S1C shows the fluorescence decay traces from cells which were first adapted to state I (red line) and then state II (black line) and finally back to state I (blue line). Decay traces which are shown in red and blue are very similar, reflecting that the state transitions were reversible in our samples. We used 440 nm pulses to selectively excite the photosystems (Chl *a*); however the light of similar wavelengths (blue light) is used to bring the cells in state I. In order to ensure that cells adapted to state II remain in state II when 440 nm excitation light is used, a decay trace at the beginning of the laser scanning (black line in Fig. S1D) was compared with the trace after 1 min of scanning (red line in Fig. S1D). Both traces are very similar, showing that cells were stable in state II for the laser scanning parameters used in this work. Both decay traces were measured for 1 sec. In our measurements, the same cells were scanned for a total time of ~2 minutes (1 min for state I plus 1 min for state II). Fig. S1E shows the decay traces measured of light-adapted cells at the beginning of the scanning (black line), after 1 min of scanning (red line) and after 2 min of scanning (blue line). All these traces were found to be almost identical, showing that there was no detectable photodamage in the cells during the measurements.


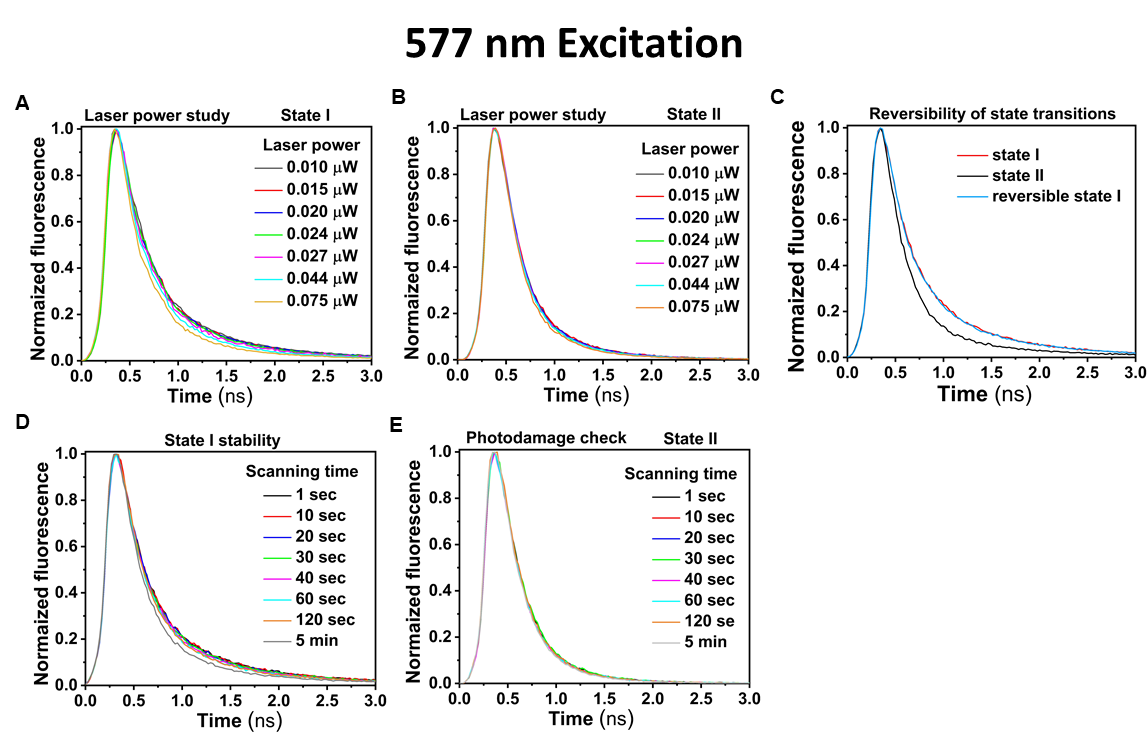


**Supplemental Figure S2.** **Fluorescence kinetics at 577 nm laser scanning.** Fluorescence decay traces of *S. elongatus* cells as a function of power of excitation pulses for cells in state I (A) and state II (B). (C) Decay traces from cells equilibrated in state I at various times of laser scanning. (D) Decay traces from cells equilibrated in state II at various times of laser scanning. (E) Decay traces from cells equilibrated in state I (red solid line), cells brought from state I to state II (black line). Cells brought back to state I (blue line). 577 nm excitation pulses were used and fluorescence was detected at 685-720 nm. All measurements were performed at room temperature. Cells were adapted for 30 minutes to blue light to induce state I and were adapted to darkness for the same time to induce state II.

In Fig. S2A fluorescence kinetics for excitation powers of 0.010 µW to 0.020 µW are very similar for state I cells. At 0.024 µW fluorescence decay started to become slightly faster (green line) and was significantly faster at laser powers of 0.044 µW (cyan line) and 0.075 µW (orange line). In Fig. S2B fluorescence kinetics were very similar from 0.010 µW to 0.027 µW and became a little faster at excitation powers of 0.044 µW and 0.075 µW. In both cases (Fig. S1A and S1B) fluorescence kinetics did not slow down when excitation power was increased from 0.01 µW showing that PSII reaction centres were closed at all powers tested. All further measurements at 577 nm were performed at 0.01 µW laser power. Fig. S1C shows the fluorescence decay traces from cells which were first adapted to state I (red line) and then to state II (black line) and finally back to state I (blue line). Decay traces which are shown in red and blue are very similar, reflecting that the state transitions were reversible in our samples. For the preferential excitation of PBSs, 577 nm pulses were used; however the light of similar wavelengths (yellow-orange) can bring the cells in state II. To ensure that, cells adapted to state I remained in state I when 577 nm excitation was used, decay traces at various times of laser scanning were compared. Fig. S1D shows that decay traces measured at the beginning of laser scanning (black line) until 30 seconds of scanning (green line) are very similar. After 40 seconds of laser scanning fluorescence decay became noticeably faster (magenta line) and a significant fastening in fluorescence decay was observed only after 5 minutes of scanning (grey line). All further measurements at 577 nm excitation were done with 20 seconds of laser scanning. Fig. S1E shows that in state II cells, fluorescence decay traces at the beginning of laser scanning (black line) until 5 minutes of scanning (grey line) are very comparable, showing that laser power did not result in detectable photodamage to the samples.

**
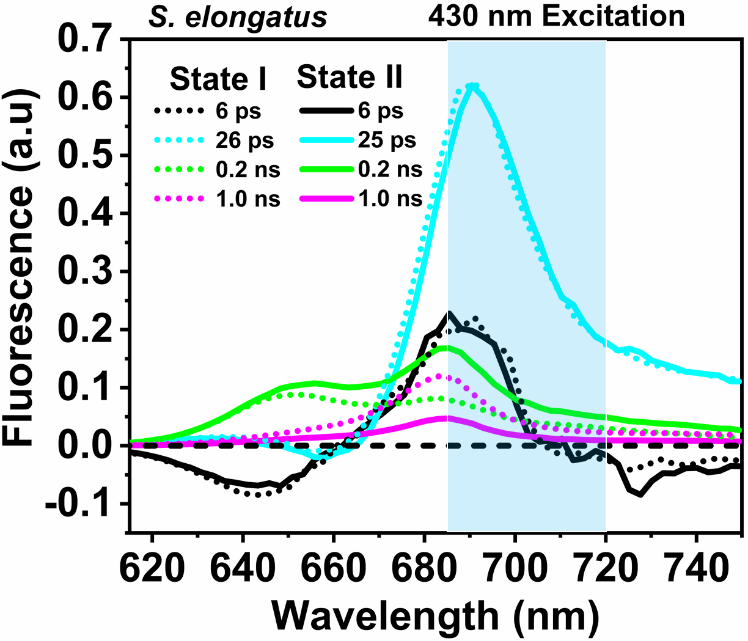
**

**Supplemental Figure S3.** **Time-resolved fluorescence spectroscopy on *S. elongatus* cells at 440-nm excitation.** Decay-associated spectra (DAS) obtained from global fitting of time-resolved fluorescence results upon 430 nm excitation of *S. elongatus* cells in state I (dotted lines) and state II (solid lines). The slowest lifetime in each case was fixed to 1.0 ns. The ~20 ps DAS in each case is multiplied with 0.5 to make the two slowest DAS better visible. DAS-associated lifetimes are shown in the inset with corresponding colours. The blueish rectangle shows the fluorescence detection region for FLIM measurements in this work. The figure is reproduced from our previous work (Bhatti et al., 2020), published under CC-BY-4.0 licence.


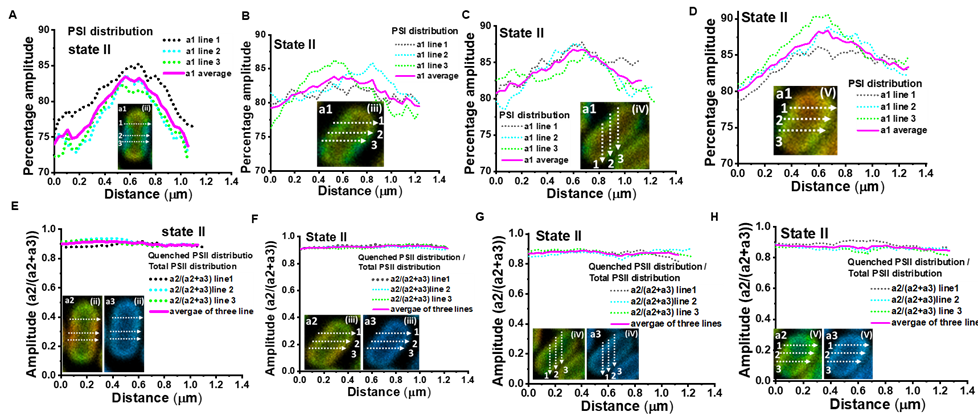


**Supplemental Figure S4.** **Profiles of PSI distribution and PSII quenching across the width of *S. elongatus* cells.** (A,B,C,D) Profile of PSI percentage acquired across the width of the cells “ii-v” (marked with white rectangles in Fig. 2) along three lines (dotted black, dotted green and dotted cyan). Average of 3 lines is shown with solid magenta line. (E,F,G,H) Ratio of quenched PSII/total PSII (a2/(a2+a3) acquired across the width of the cells “ii-v” (marked with white rectangles in Fig. 2) along three lines (dotted black, dotted green and dotted cyan). Average of thrree lines is shown with the solid magenta line. Cells “ii-v” are shown in inset.


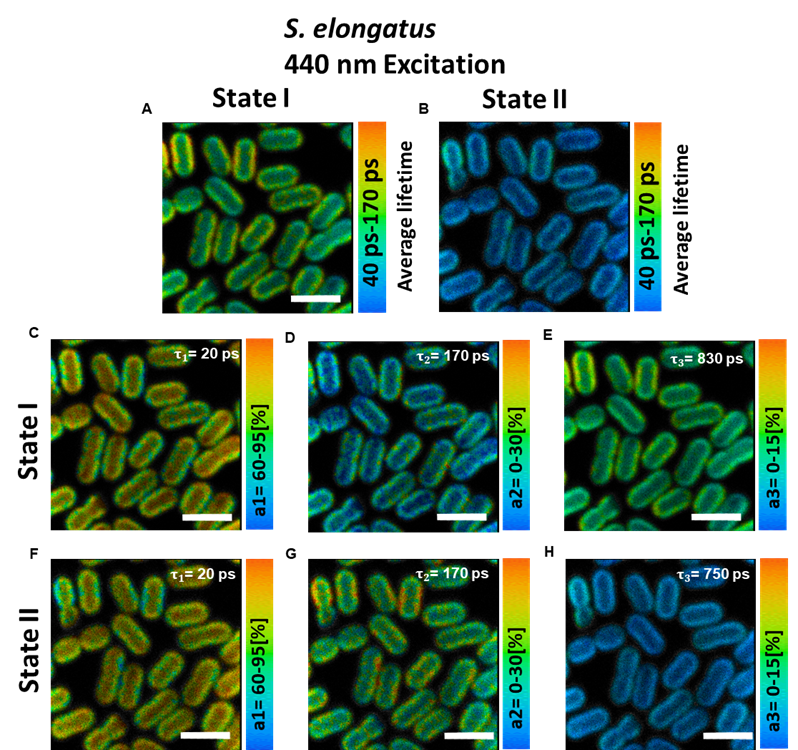


**Supplemental Figure S5.** **FLIM analysis of *S. elongatus* cells at low pixel binning.** (A,B) Average fluorescence lifetime distributions in state-I and state-II adapted cells. Distribution of amplitudes associated with the fluorescence lifetimes in *S. elongatus* cells in state I (C,D,E) and state II (F,G,H). Corresponding lifetimes are written in the figures. Same cells were imaged for both state I and State II measurements. Excitation wavelength was 440 nm and fluorescence was recorded in 685 – 720 nm range. Scale bar is 4 µm. Images are adjusted for brightness and contrast. Measurements were performed at room temperature.

In Fig. 1 and Fig. 2, fluorescence lifetime analysis was performed at pixel binning of 7 to achieve better signal/noise ratio for the estimation of fit parameters (lifetimes and amplitudes). The lifetimes found with this analysis were fixed and pixel binning was reduced to 2. Fig. S8 shows the average fluorescence lifetime images (A,B) and amplitude profiles associated with the lifetimes (C-H) at the reduced pixel binning of 2. Comparison of supplemental figure. S5 with the Fig. 1 and Fig. 2 shows that the results obtained at lower and higher binning are very similar. At binning 2, slightly broader distribution of amplitudes was observed as compared to binning 7. A high amplitude of ~20 ps component (representing PSI) in the centre of the cells can therefore not be ascribed as an artefact of pixel averaging at higher binning.


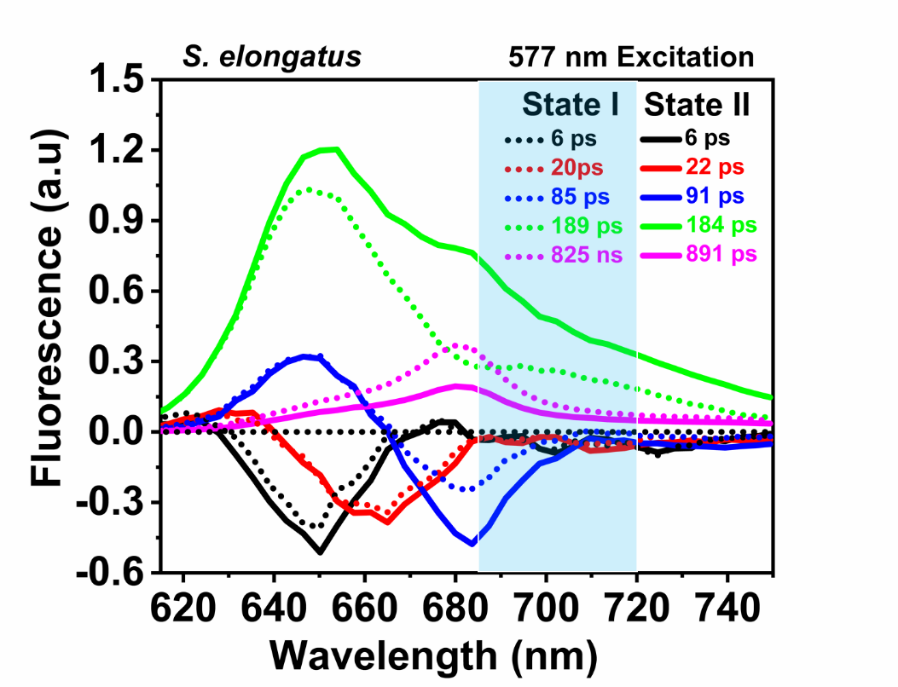


**Supplemental Figure S6.** **Time-resolved fluorescence spectroscopy on *S. elongatus* cells at 577-nm excitation.** Decay-associated spectra obtained from global fitting of time-resolved fluorescence results after 577 nm excitation of *S. elongatus* cells in state I (dotted lines) and state II (solid lines). DAS-associated lifetimes are presented in the inset with corresponding colours. The bluish rectangle shows the fluorescence detection region for FLIM measurements in this work. The figure is reproduced from our previous work (Bhatti et al., 2020), published under CC-BY-4.0 licence.


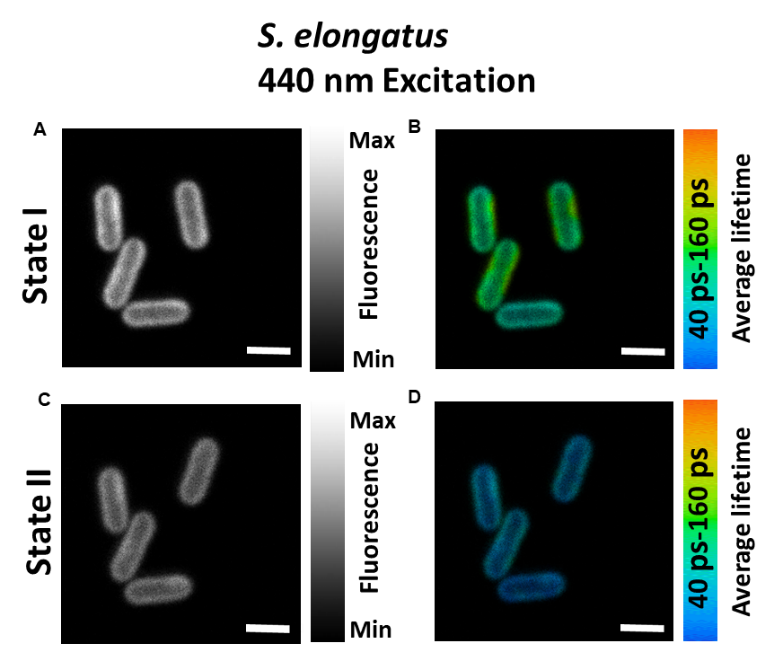


**Supplemental Figure S7.** **At 440-nm excitation confocal fluorescence microscopy images of *S. elongatus* cells adapted to state I and state II.** A, C show steady-state intensity images of cells in state I and state II, respectively. (C,D) show average lifetime distributions in state-I and state-II adapted cells. Same cells were imaged for both state I and State II measurements. Excitation wavelength was 440 nm and fluorescence was recorded in 685 – 720 nm range. Scale bar is 2 µm. Images are presented without adjustments for brightness and contrast. Measurements were performed at room temperature.

**
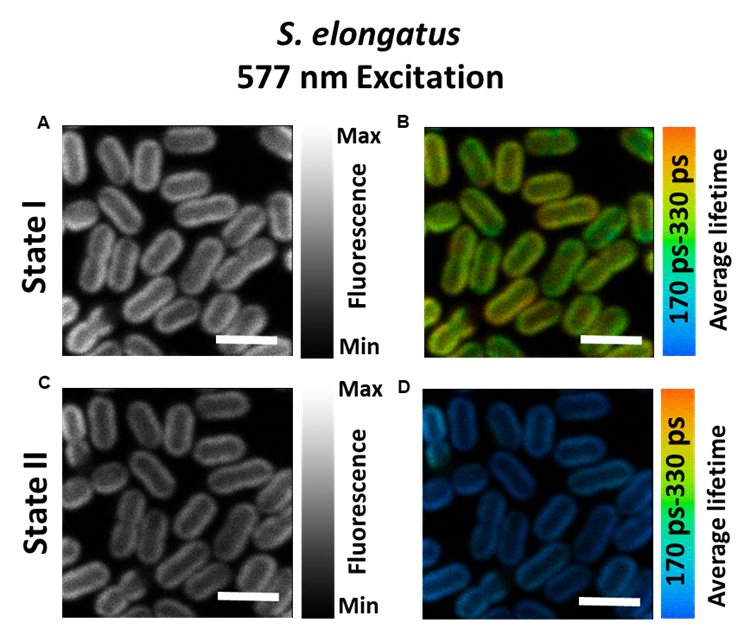
**

**Supplemental Figure S8.** **At 577-nm excitation confocal fluorescence microscopy images of *S. elongatus* cells adapted to state I and state II.** A, C show steady-state intensity images of cells in state I and state II, respectively. (C,D) show average lifetime distributions in state-I and state-II adapted cells. Same cells were imaged for both state I and State II measurements. Excitation wavelength was 577 nm and fluorescence was recorded in 685 – 720 nm range. Scale bar is 4 µm. Images are presented without adjustments for brightness and contrast. Measurements were performed at room temperature.
